# Supplementary material for: Measurement matters: changing penalty calculations under the hospital acquired condition reduction program (HACRP) cost hospitals millions
Source: BMC Health Serv Res. 2021 Feb 10;21:131. doi: 10.1186/s12913-021-06108-w (PMC7874626; doi:10.1186/s12913-021-06108-w)
Supplement: Supplementary file 1 — Additional file 1 Online Appendix. [file 12913_2021_6108_MOESM1_ESM.pdf]

Online Appendix

Measurement Matters: Changing Penalty Calculations under the Hospital Acquired Condition Reduction Program (HACRP) Cost Hospitals Millions

Table A1. Descriptive statistics of total HAC scores

	No data submitted to NHSN implies no Domain 2 score		No data submitted to NHSN implies maximum Domain 2 score		
	Mean (SD)	75th percentile cutoff	Mean (SD)	75th percentile cutoff	CMS reported 75th percentile cutoff
FY 2018	-0.06 (0.72)	0.263	0.94 (1.12)	1.999	0.371
FY 2017	5.72 (2.23)	7.000	7.31 (2.56)	9.400	6.570
FY 2016	5.91 (2.32)	8.000	7.48 (2.35)	9.250	6.750
FY 2015	5.85 (2.38)	8.000	7.25 (2.31)	8.950	7.000

Source: Author's analysis of HCUP administrative data and NHSN HAIs for hospitals in 14 states.

Figure A2. Changes in total HAC scores by FY-specific methodology.

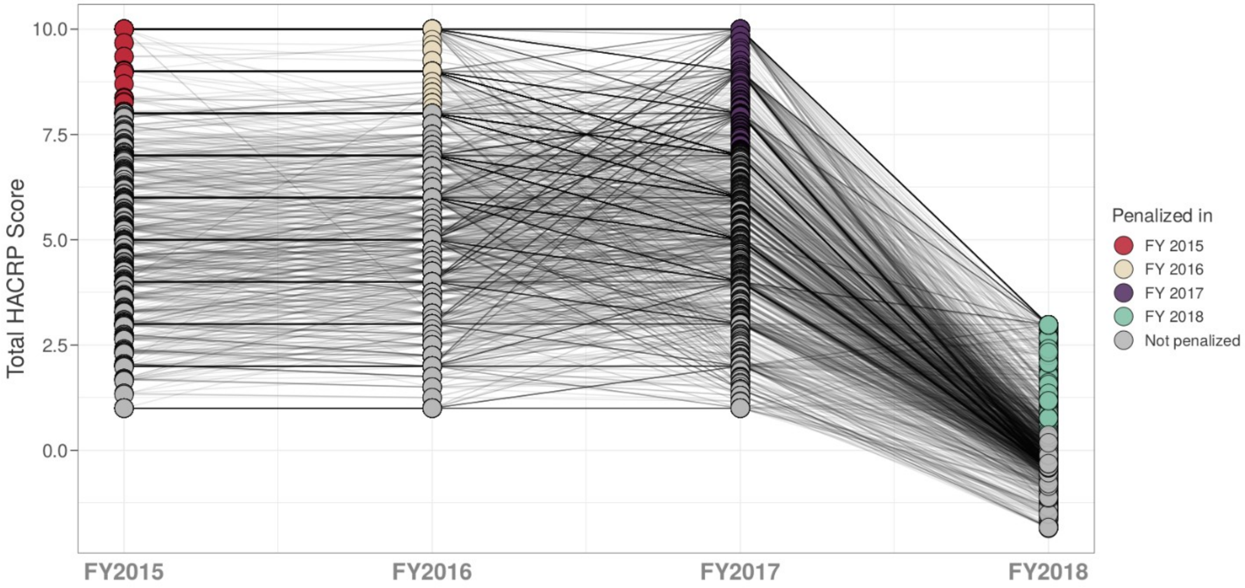

Source: Author's analysis under the assumption that if no HAI data was submitted to NHSN then no Domain 2 score.

Notes: Points in color represent total HAC scores that are greater than the 75th percentile of the empirical distribution.

Figure A3. Percent overlap in penalized hospitals among FY 2015-2018 scoring methodologies.

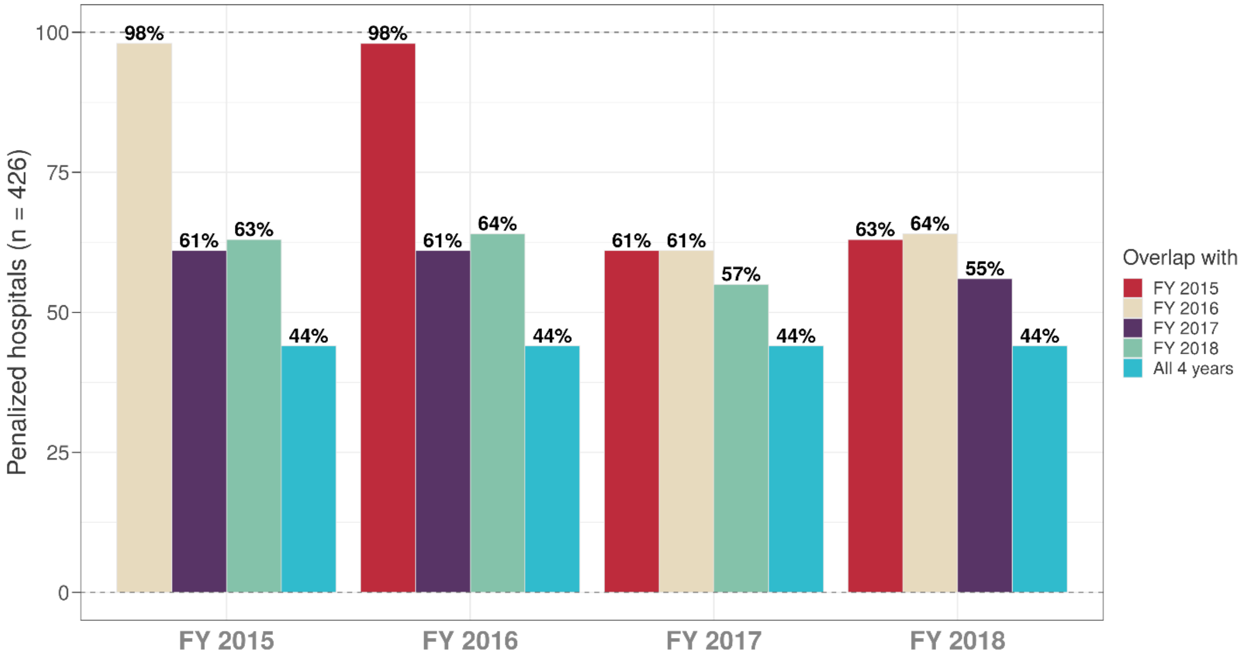

Source: Author's analysis under the assumption that if no HAI data was submitted to NHSN then maximum Domain 2 score.

Figure A4. Changes in total HAC scores by FY-specific methodology.

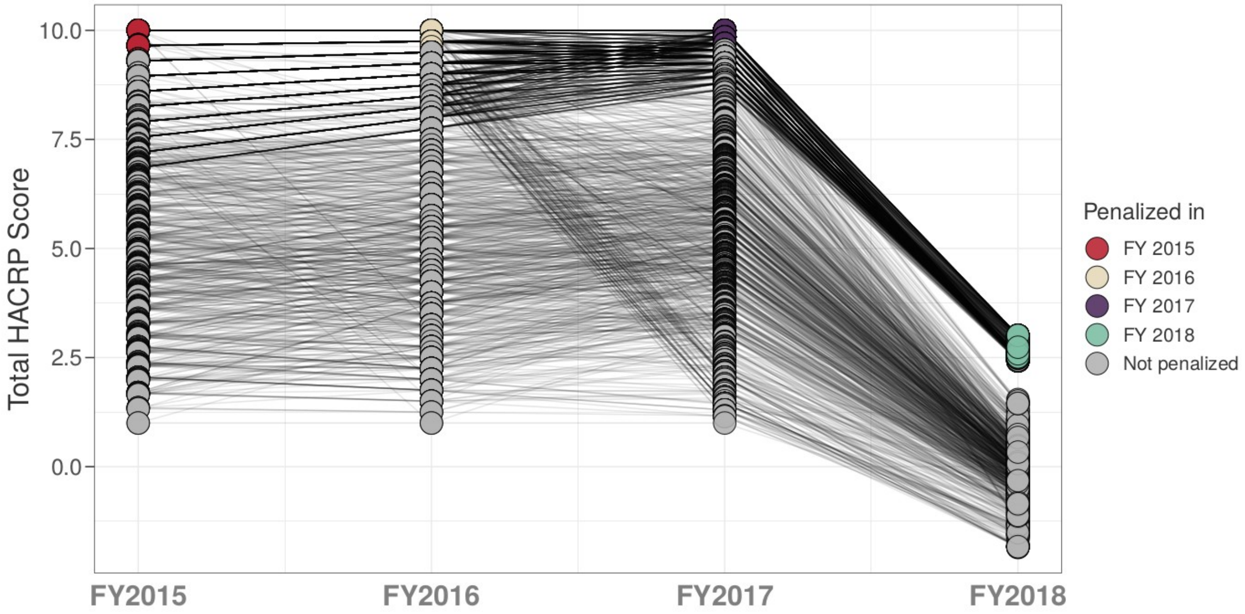

Source: Author's analysis under the assumption that if no HAI data was submitted to NHSN then maximum Domain 2 score.

Notes: Points in color represent total HACRP scores that are greater than the 75th percentile of the empirical distribution.
